# Tacrine-6-Ferulic Acid, a Novel Multifunctional Dimer, Inhibits Amyloid-β-Mediated Alzheimer's Disease-Associated Pathogenesis *In Vitro* and *In Vivo*

**DOI:** 10.1371/journal.pone.0031921

**Published:** 2012-02-23

**Authors:** Rongbiao Pi, Xuexuan Mao, Xiaojuan Chao, Zhiyi Cheng, Mengfei Liu, Xiaolu Duan, Mingzhong Ye, Xiaohong Chen, Zhengrong Mei, Peiqing Liu, Wenming Li, Yifan Han

**Affiliations:** 1 Department of Pharmacology and Toxicology, School of Pharmaceutical Sciences, Sun Yat-Sen University, Guangzhou, China; 2 Department of Neurology, The Third Affiliated Hospital, Sun Yat-Sen University, Guangzhou, China; 3 Department of Applied Biology and Chemical Technology, Institute of Modern Chinese Medicine, The Hong Kong Polytechnic University, Hong Kong, China; 4 Department of Pharmacology and Neurology, School of Medicine, Emory University, Atlanta, Georgia, United States of America; Indiana University School of Medicine, United States of America

## Abstract

We have previously synthesized a series of hybrid compounds by linking ferulic acid to tacrine as multifunctional agents based on the hypotheses that Alzheimer's disease (AD) generates cholinergic deficiency and oxidative stress. Interestingly, we found that they may have potential pharmacological activities for treating AD. Here we report for the first time that tacrine-6-ferulic acid (T6FA), one of these compounds, can prevent amyloid-β peptide (Aβ)-induced AD-associated pathological changes *in vitro* and *in vivo*. Our results showed that T6FA significantly inhibited auto- and acetylcholinesterase (AChE)-induced aggregation of Aβ_1–40_ *in vitro* and blocked the cell death induced by Aβ_1–40_ in PC12 cells. In an AD mouse model by the intracerebroventricular injection of Aβ_1–40_, T6FA significantly improved the cognitive ability along with increasing choline acetyltransferase and superoxide dismutase activity, decreasing AChE activity and malondialdehyde level. Based on our findings, we conclude that T6FA may be a promising multifunctional drug candidate for AD.

## Introduction

Alzheimer's disease (AD) is a multifactorial neurodegenerative disorder with progressive and devastating memory impairment [1]. The AD patient brain is characterized by amyloid-β peptide (Aβ) deposits, neurofibrillary tangles, synapse loss, and extensive oxidative stress. Aβ-induced oxidative stress is indexed by protein oxidation, lipid peroxidation, free radical formation, DNA oxidation and neuronal cell death [2]–[3]. The majority of therapeutic strategies and drug development approaches for AD were based on dysfunction of acetylcholine to date, which mainly improves the pathological symptom [4]. Inhibition of Aβ fibril aggregation and antioxidants are also viewed as promising strategies to halt the progression of AD [5]–[8]. Unfortunately, current “one-molecule-one-target” drugs are not effective strategy to delay or block the progress of AD pathology because of multiple causes, such as cholinergic deficiency, Aβ and tau protein toxicity, oxidative stress and so on. Now, “one-compound-multi-targets” strategy, which simultaneously aimed at targeting multiple pathological processes, gradually shows its potential advantages [9]–[10].

**Figure 1:**
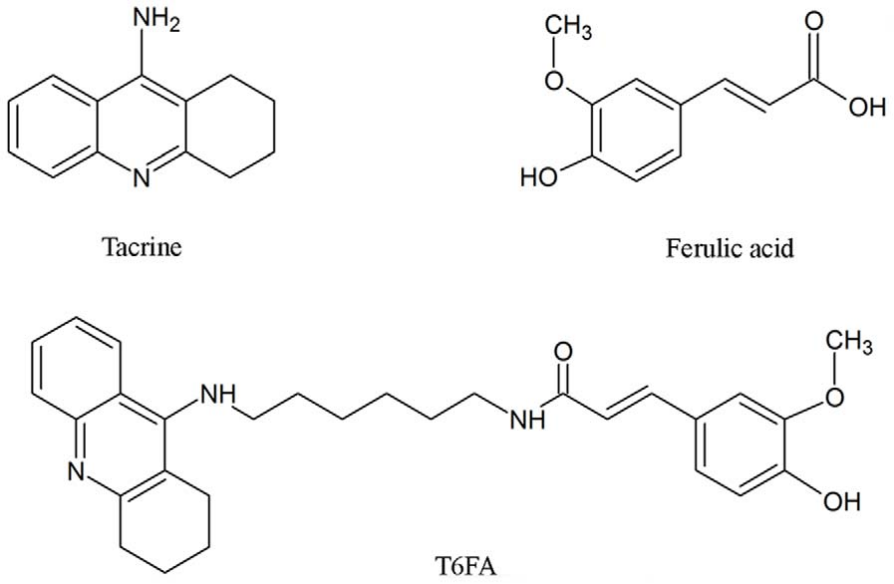
Chemical structures of tacrine, ferulic acid, and the hybrid moleculeT6FA.

Tacrine (1, 2, 3, 4-tetrahydro-9-acridinamine, THA, **1**, Figure 1) is the first centrally acting cholinesterase inhibitor to be widely applied for the loss of memory and intellectual decline in patients of AD. Though some deficiencies of tacrine emerged gradually including hepatotoxic effect and low-selective peripheral cholinergic effect, recent studies have demonstrated its homo- and hetero-dimers can improve and enlarge its biological profile with less side-effects [9], [11]. Ferulic acid (4-hydroxy-3- methoxycinnamic acid, FA, **2**, Figure 1), a bioactive component of Traditional Chinese Medicine, has antioxidant and anti-inflammatory effects [12], inhibits Aβ fibril aggregation [13], and prevents Aβ-mediated toxicity both *in vitro* and *in vivo* [14]–[15]. Besides these benefits in the central nervous system, FA also possesses hepatoprotective effects which may prevent the hepatotoxic effect of tacrine [16].

Previously, Fang *et al* and we synthesized and evaluated a series of tacrine-ferulic acid hybrids as multipotent anti-AD drug candidates [17]–[21]. These compounds have better acetylcholinesterase (AChE) inhibitory activity and comparable butyrylcholinesterase (BuChE) inhibitory activity in relation to tacrine [17], [18]. In addition, these compounds can also inhibit 1,1-Diphenyl-2-picrylhydrazyl (DPPH) radical formation [17], [19]. Some of them have been proved to reverse scopolamine-induced cognitive impairment in mice or rats with low hepatotoxicity [18]–[20]. Interestingly, novel ferulic acid and benzothiazole dimer derivatives have been found to specifically bind to Aβ fibrils (fAβ) and to inhibit fibril aggregation as FA [22]–[23]. These results suggest that tacrine-ferulic acid hybrids might also inhibit the fibril aggregation of Aβ and Aβ-mediated toxicity, potential targets for AD therapy [5]–[8].

In this study, we examined the effects of tacrine-6-ferulic acid (T6FA, Figure 1), a novel tacrine-ferulic acid dimer, on Aβ aggregation, Aβ-induced cell death *in vitro* and cognitive impairment in a mouse model of AD induced by aggregated Aβ.

## Materials and Methods

### Materials

Aβ_1–40_, the reverse sequences Aβ_40–1_, Tacrine hydrochloride, Donepezil hydrochloride, Human acetylcholinesterase (HuAChE) and 3-(4,5-dimethylthiazol-2-yl)-2,5- diphenyltetrazolium bromide (MTT) were purchased from Sigma Chemical Co. (agency in China). 2,7-dichlorofluoroescin diacetate (H_2_DCF-DA) were purchased from Molecular Probes Co. (agency in China). Tacrine-6-ferulic acid (purity >98%) was synthesized by our laboratory [17]. RPMI 1640 medium, antibiotics (penicillin/streptomycin), horse serum and fetal bovine serum were purchased from Invitrogen (Grand Island, NY, U.S.A.). Each test compound (tacrine, T6FA and ferulic acid) at a concentration of 50 mM was dissolved in DMSO and further diluted with PBS. The final concentration of DMSO was no more than 0.1% in the medium, which did not affect cell viability. A fresh 10 mM stock solution of H_2_DCF-DA was prepared in ethanol. Fresh T6FA (1 mg/mL) was prepared in phosphate-buffered saline. The cells were pre-incubated with 2–50 µM T6FA for 30 min before the addition of 20 µM of Aβ_1–40_. The commercial kits for the assay of AChE (Cat #, A024), choline acetytransferase (Cat #, A079), superoxide dismutase (Cat #, A001-1) and malondialdehyde (Cat #, A003-2) were purchased from Nanjing Jiancheng Biotech Company, China (www.njjcbio.com).

### Assay of AChE-induced Aβ aggregation [24]

Aliquots of 2 µL Aβ_1–40_, lyophilised from 2 mg/mL hexafluoroisopropanol solution and dissolved in DMSO, were incubated for 48 h at room temperature in 0.215 M sodium phosphate buffer (pH 8.0) at a final concentration of 230 µM. For co-incubation experiments aliquots (16 µL) of HuAChE (final concentration 2.30 µM, Aβ/AChE molar ratio 100∶1) and HuAChE in the presence of 2 µL of the tested inhibitors in 0.215 M sodium phosphate buffer pH 8.0 solution (final inhibitor concentration ranging between 50 and 100 µM) were added. Blanks containing Aβ, HuAChE, and Aβ plus inhibitors at various concentrations in 0.215 M sodium phosphate buffer (pH 8.0) were prepared. The final volume of each vial was 20 µL. Each assay was run in duplicate. To quantify amyloid fibril formation, the thioflavin T fluorescence method was then applied. After incubated at 37°C for 24 h, the solutions containing Aβ, or Aβ plus AChE, or Aβ plus AChE in the presence of inhibitors were added to 50 mM glycine–NaOH buffer (pH 8.5) containing 1.5 µM thioflavin T in a final volume of 2.0 mL. Fluorescence was monitored with excitation at 446 nm and emission at 490 nm soon after the solution was mixed. The fluorescence intensities were compared and the percent inhibition due to the presence of test compounds was calculated. The percent inhibition of the HuAChE-induced aggregation due to the presence of increasing test compound concentration was calculated by the following expression: 100−(IF_i_/IF_o_×100) where IF_i_ and IF_o_ are the fluorescence intensities obtained for Aβ plus HuAChE in the presence or absence of inhibitor, respectively. Inhibition curves were obtained for each compound by plotting the percentage inhibition versus the logarithm of inhibitor concentration in the assay sample. The linear regression parameters were determined and the IC_50_ extrapolated, when possible (GraphPad Prism 3.0 GraphPad Software Inc.).

### Transmission electron microscopy (TEM) to assay the fAβ

Assay of Aβ_1–40_ fibril formation using TEM images as previously described [25]. Briefly, TEM analysis was performed to observe size and structural morphology changes of fAβ_1–40_ in the presence or absence of T6FA or FA at different concentrations. A mixture of freshly prepared Aβ_1–40_ solution (10 µL of 50 µM in 10 mM sodium phosphate at pH 7.4) was incubated for 72 h at 37°C. The TEM samples were prepared by placing 5 µL of the pre-incubated solution on a carbon-coated grid. The samples were stained with 1% uranyl acetate and were placed on a clean paper for removing excess staining solution. The grids were thoroughly examined using a Phillips CM-30 electron microscope. Images were recorded at 52,000× magnification in a Philips/FEI CM120 electron microscope with a Gatan GIF100 imaging filter, equipped with a cooled slow scan CCD camera.

### Cell Culture and Treatment

PC12 cells originally obtained from American Tissue Type Cell Collection (ATCC) were grown in RPMI 1640 medium supplemented with 10% horse serum, 5% fetal bovine serum, and 1% antibiotics (penicillin/streptomycin) at 37°C in a humidified 95% air/5% CO_2_ incubator. Cells were pretreated with test compound and incubated for 30 min. After that, Aβ_1–40_ solution (final concentration, 20 µM) was added to culture medium and incubated for 24 h. Controls were only treated with the vehicle or with the reverse peptide Aβ_40–1_ (final concentration, 20 µM). The reverse sequence Aβ_40–1_ was prepared in the same way of that of Aβ_1–40_.

### Cell viability Assays

Assays for cell viability were performed after 24 h of Aβ_1–40_ or Aβ_40–1_ treatment as previously described [26]. Photomicrographs were taken with a camera attached to microscope (Olympus, Japan) after 24 h of treatment to assess morphological alterations. Cell viability was assessed by measuring formazan produced by the reduction of MTT. PC12 cells in 48-well culture dishes were treated with Aβ and incubated for 24 h at 37°C. Briefly, after treatment for 24 h, MTT solution (final concentration, 500 µg/mL) was added and cells were incubated at 37°C for 1 h. After this, the medium was removed and the cells were solubilized with dimethylsulfoxide and transferred to a 96-well plate. The formazan reduction product was measured by reading absorbance at 560 nm in a plate reader (BioTek, China).

### Reactive oxygen species (ROS) detection

Intracellular ROS formation was measured by fluorescence using H_2_DCF-DA [27]. Briefly, PC12 cell cultures grown on 96-well plates were incubated with 10 µM H_2_DCF-DA (Molecular Probes, agency in China) for 30 min at 37°C after 24 h of Aβ_1–40_ treatment in the presence or absence of T6FA. The cells were then rinsed with PBS solution. Intracellular esterases convert DCF diacetate to anionic DCFH which is trapped in the cells. The fluorescence of DCF, formed by the reaction of DCFH with ROS was recorded (504 nm excitation, 529 nm emission) using a PerkinElmer LS-5B spectrofluorometer.

### Animals

The animal experiments were performed according to internationally followed ethical standards and approved by the research ethics committee of Sun Yat-sen University (No 20081102). The investigation conformed to the Guide for the Care and Use of Laboratory Animals published by the US National Institutes of Health (NIH Publication No. 85-23, revised 1996). Specific pathogen free SPF C57 BL/6J mice (male, weighing 18–20 g) were supplied by the Experimental Animal Center of Sun Yat-sen University (Guangzhou, China) and housed in separate cages in standard conditions. Animals were housed in standard laboratory conditions: air-conditioned room (20–25°C), 12 h light/dark illumination cycle, free access to food and water.

### Aβ intracerebroventricular (icv) AD model and drugs administration

**Figure 2:**
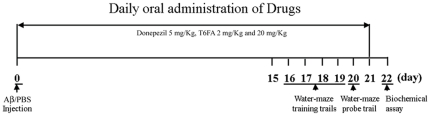
Experimental schedules.

Aβ_1–40_ was prepared as stock solution at a concentration 0.6 µg/µL in sterile 0.1 M phosphate-buffered saline (PBS) (pH 7.4), and aliquots were stored at −20°C. Aβ_1–40_ was aggregated by incubation in sterile distilled water at 37°C for 4 d before use as described previously [28]. Aβ_1–40_ (400 pmol/mouse) or PBS was administered by icv route using a microsyringe (10 µL, Hamilton) that was inserted perpendicularly 3 mm deep through the skull. Briefly, the C57 BL/6J mice were anesthetized with 10% chloral hydrate (3.5 ml/kg body weight) dissolved in saline and then 3 µL of Aβ or sterile PBS was injected directly into the lateral ventricle, at the following coordinates from bregma taken from the atlas of mouse [11]: anterioposterior (AP) = −0.1 mm; mediolateral (ML) = 1 mm; and dorsoventral (DV) = −3 mm. 1 mg T6FA was dissolved in 0.15 mL PEG-400 and diluted by 0.35 mL saline for T6FA (20 mg/kg), then diluted to one tenth by vehicle (PEG-400∶saline = 3∶7) for T6FA (2 mg/kg). After surgery, each mice was randomly assigned to one of five groups (*n* = 10) to receive an intragastric administration of vehicle (Sham and Model), T6FA (2 mg/kg and 20 mg/kg), Donepezil (5 mg/kg) for consecutively 21 d. The protocol was outlined in Figure 2.

### Morris water maze (MWM) test

The testing procedure was carried out as described previously [29]. The maze consists of 1 m diameter black circular pool with side walls 30 cm high. The pool is filled with water at a temperature of 21∼25°C to a depth of 20 cm. The platform is hidden approximately 1 cm below the surface of the water in the 3rd quadrant. 16 d after Aβ_1–40_ administration, five groups were subjected to the MWM as described. Prior to first trial of each day, mice were placed on platform for 30 s for spatial orientation. Then mice were placed in a random start site, facing tank wall, initially mice were trained for 4 d for hidden platform trial, at 4th day were trained for probe trial after hidden platform trial. The escape latency is recorded as a parameter for hidden platform trial, and the time spent in goal area is recorded as a parameter for probe trial.

### Measurement of choline acetyltransferase (ChAT) and AChE activity

Following MWM test, animals of each group were decapitated under anesthesia at d22 after Aβ_1–40_ administration. The brains were removed quickly and the hippocampi were dissected on ice. Samples were weighed and homogenized to 10% homogenate (100 mg sample in 1 mL ice-cold saline) for AChE. Aliquote of the homogenate were further diluted with saline to 5% for ChAT assay. The supernatant was used to determine AChE and ChAT activity respectively. Protein concentration was determined by the method of BCA. The activities of ChAT and AChE were determined spectrophotometrically using the assay kit from Nanjing Jiancheng Bioengineering Institute (Nanjing, China). The absorbance was read at 324 nm, and ChAT activity was expressed as unite per g protein. For the assay of AChE activity, a reaction mixture that contained sodium phosphate (1 mM, pH 8.0) 470 µl, 2% DTNB 167 µl and 33 µl of homogenate was incubated for 5 min at 37°C. Then, acetylcholine iodide (2 mM) 280 µl was added to the reaction mixture. After incubation for 3 min at 37°C, the reaction was terminated by adding 50 µl of neostigmine (4 mM). The absorbance was measured at 412 nm at room temperature. AChE activities were expressed as µmol per µg of protein.

### Measurement of superoxide dismutase (SOD) activity and malondialdehyde (MDA) level

MDA level and SOD activity were detected using commercial kits (Nanjing Jiancheng Biotech., China) in tissue homogenates diluted to 10% and prepared in accordance with the manufacturer's instructions. The 10% homogenate made as described before was also used here for MDA assay and diluted with saline for the determination of SOD activity. Activity of SOD in hippocampus was measured by the method reported using nicotinamide adenine denucleotide reduced form as a substrate [30]. The SOD activity was expressed as units/mg protein. One unit of the enzyme was the amount required to inhibit the rate of chromogen formation by 50%. As a measure of lipid peroxidation, MDA levels in brain tissue were estimated by measuring thiobarbituric acid reactive substances following the standard protocol using MDA detection kit and were expressed as nmol per mg of protein (nmol per mg protein).

### Statistical Analysis

The means and standard errors of means (SEM) were calculated for all experiments. The data were subjected to one-way analysis of variance (ANOVA) followed by Duncan's multiple-range test to determine whether means were significantly different from the control or model. In all cases, a *P* value of <0.05 was accepted to determine the significance.

## Results

### Effect of T6FA on AChE-induced and auto-aggregation of Aβ

Accumulating evidence demonstrates that AChE has secondary non-cholinergic functions including the processing and deposition of Aβ [31]. AChE can accelerate Aβ deposition through its peripheral anionic site (PAS). Compounds binding to the PAS can inhibit AChE-accelerated fibril aggregation. T6FA is a novel dual-binding site AChE inhibitor which can modulate both the cholinergic and amyloid targets [17], [19], indicating that T6FA may inhibit Aβ aggregation and deposition. To assess T6FA's ability of inhibiting Aβ aggregation induced by AChE, a thioflavin T-based fluorometric assay was used [24]. As shown in Table 1, T6FA significantly inhibited the AChE-induced Aβ_1–40_ aggregation by 50.27% and 20.23% at 100 µM and 50 µM, respectively, while the effects of FA and tacrine on Aβ aggregation were lower or not detected at such concentrations.

**Table 1:** T6FA Inhibits AChE-induced Aβ aggregation.

Compound	% inhibition of Aβ Aggregation^a^	
	100 µM^b^ (%)	50 µM^c^ (%)
T6FA	50.27	20.23
1(tacrine)	8.31	NA^d^
2(ferulic acid)	28.66	7.5

a Co-aggregation inhibition of Aβ_1–40_ 20 µM and AChE 0.02 U was detected by thioflavin T assay.

b The data (%) showed that the test compounds inhibited the coaggregation at 100 µM.

c The data (%) showed that the test compounds inhibited the co-aggregation at 50 µM.

d Nd represents for “not available”.

**Figure 3:**
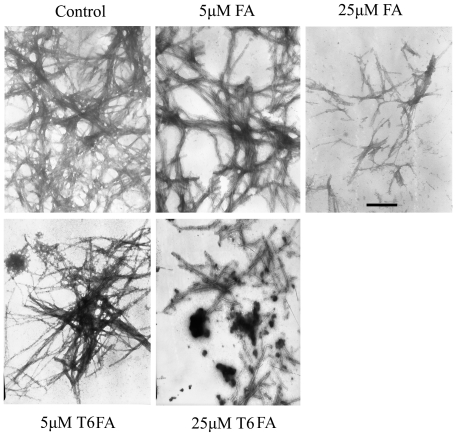
T6FA inhibits the Aβ auto-aggregation. Electron micrographs of Aβ_1–40_ were taken with or without ferulic acid (FA) and T6FA. The reaction mixture containing 25 µM Aβ_1–40_, 50 mM phosphate buffer, pH 7.5, 100 mM NaCl, and 5–25 µM FA or T6FA was incubated at 37°C for 24 h. Scale bar = 100 nm.

It was well documented that antioxidants, including FA, can inhibit Aβ auto-aggregation [13]. To further observe the effects of T6FA on the auto-aggregation of Aβ, the transmission electron microscopy (TEM) was used. Aβ became long and network fibrils after incubating for 72 h (Figure 3). The fibrils Aβ were significantly decayed when FA or T6FA (10–25 µM) was present. It is noteworthy that T6FA almost completely inhibited fibril production at 25 µM. (Figure 3).

### T6FA prevents the cell death and reduces intracellular ROS induced by Aβ_1–40_ in PC12 cells

**Figure 4:**
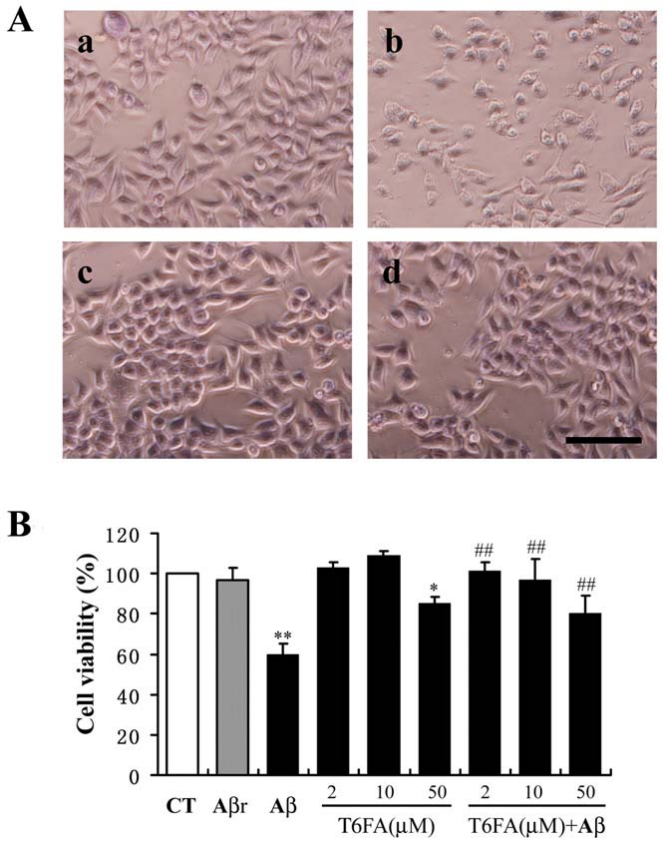
T6FA inhibits Aβ-induced cell death in PC12 cells. PC12 cells were pretreated with T6FA (2–50 µM) for 30 min, before exposure to Aβ_1–40_ (20 µM) or the reverse peptide Aβ_40–1_ or vehicle for additional 24 h. The cellular viability was evaluated by phase-contrast microscopy observation (A) and MTT assay (B). (A) Representative phase-contrast photographs of PC12 cells: a, control; b, Aβ_1–40_; c, T6FA and d, T6FA plus Aβ_1–40_. Scale bar = 50 µM. (B) The data were expressed as percentage of the control (non-treated cells). The control treatment is set to 100%. Bars are means ± S.E. We used ** P<0.01 and * P<0.05 versus control, ##P<0.01 versus Aβ_1–40_ treatment (n = 6).

Aβ-induced apoptotic neuronal cell death is a critical event in the pathology of AD. To investigate whether T6FA can attenuate the cell death induced by Aβ_1–40_, PC12 were pretreated with T6FA (2–50 µM) for 30 min and then exposed to 20 µM Aβ_1–40_ for 24 h before cell viability was assayed. Phase contrast images showed that the cells were fewer in number, less viable with shrinked cell body, many fragments, and less adhered after the addition of Aβ_1–40_. T6FA (10 µM) treatment blocked these effects of Aβ_1–40_ (Figure 4A). Cell viability assay inT6FA dramatically prevented the cell death induced by Aβ_1–40_ in PC12 cells even in 2 µM, while the reverse sequence of Aβ_40–1_ had no effect on the cell viability (Figure 4B). Although inducing some toxicity at 50 µM, T6FA still attenuated the Aβ_1–40_-mediated cell death at such concentration (Figure 4B).

**Figure 5:**
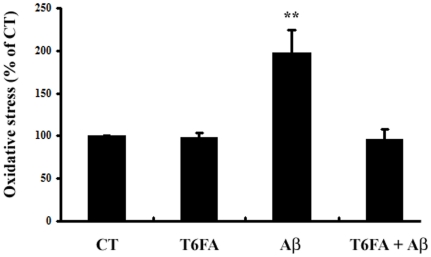
T6FA inhibits Aβ_1–40_-induced intracellular ROS accumulation in PC12 cells. Treatment of cells and measurement of oxidative stress levels using the DCF-DA assay were as described in Materials and Methods section. The data were expressed as the mean ± S.E.M. as a percentage of control values. Statistical comparison was analyzed by ANOVA (*n* = 5). **P<0.01, Aβ_1–40_ versus Control.

Oxidative stress plays a pivotal role in the progression of AD and Aβ can induced neuronal cell death and intracellular ROS accumulation [3]. In present study, Aβ_1–40_ (20 µM) increased the intracellular ROS accumulation by about 2.0-folds after 24 h in the PC12 cells. Treatment of T6FA (10 µM) profoundly reduced the Aβ_1–40_-induced production of ROS to basal levels without affecting basal levels of ROS (Figure 5).

### Effect of T6FA on the cognitive impairment in Aβ icv mice

**Figure 6:**
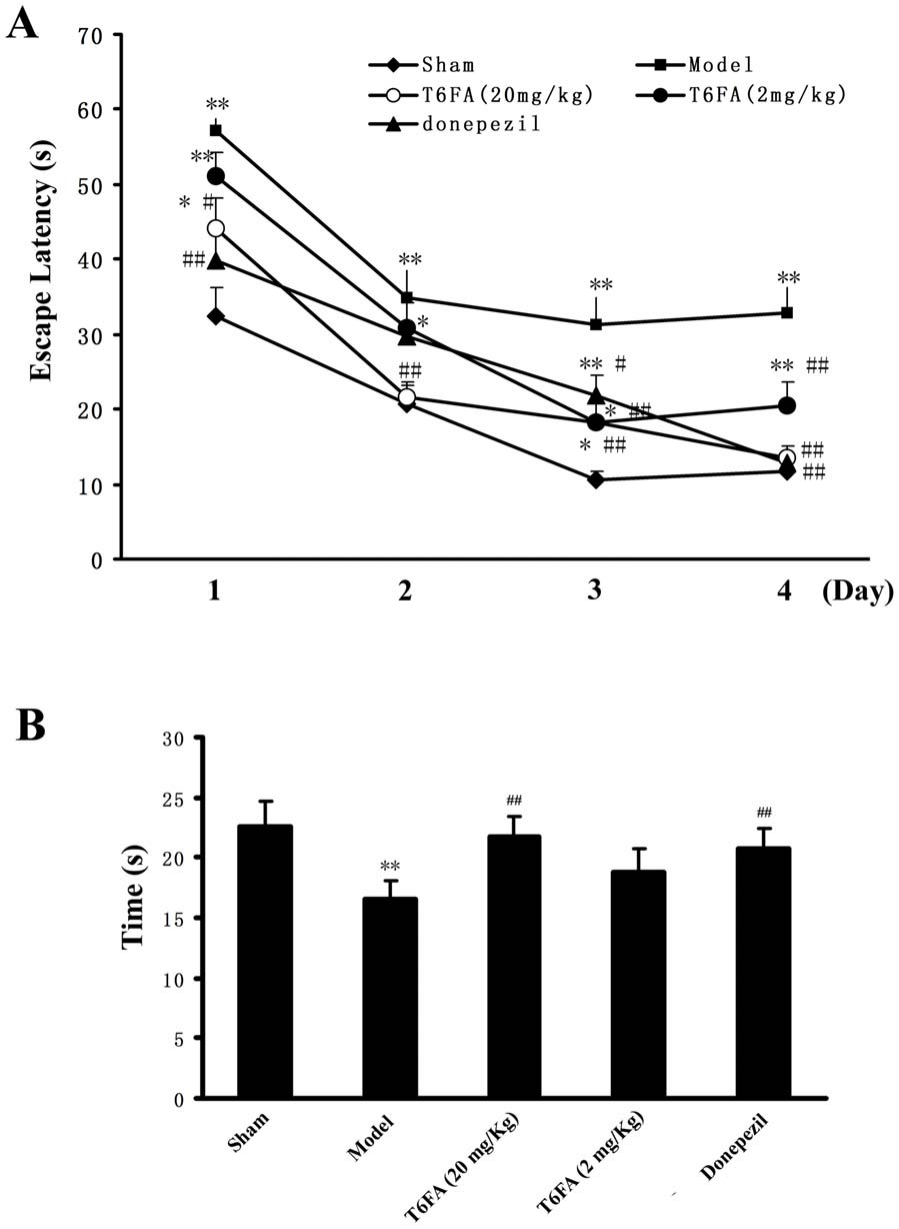
T6FA improves the cognitive impairment in Aβ_1–40_-injected mice. Aβ_1–40_ (400 pmol/mouse) or PBS was administered by icv route using a microsyringe. After surgery, each mouse received an intragastric administration of vehicle (Sham and Model), T6FA (2 mg/kg and 20 mg/kg), Donepezil (5 mg/kg) for consecutive 21 d. The MWM test was used to observe the spatial learning and memory performance of mice at d16 after surgery. Escape latencies in hidden-platform (A) and spatial preference pattern in a probe test (B). T6FA treatment could significantly shorten the escape latency and increase the swimming time in object quadrant compared with model mice. Data were presented as mean ± S.E.M. (*n* = 10). *P<0.05, **P<0.01 versus sham-operated group; ^#^ *P*<0.05, ^# #^ *P*<0.01 versus model group.

The effect of T6FA (2 mg/kg, 20 mg/kg, i.g.) on spatial learning was evaluated by MWM test in an AD model in mice. As shown in Figure 6 A, the mice in model group exhibited longer escape latency than that in sham-operated group during the test (*P*<0.01). The increased escape latencies could be significantly attenuated by Donepezil (5 mg/kg) at d1, d2 and d4 (*P*<0.05). T6FA (20 mg/kg) could markedly shorten the increased escape latencies and such effects were mostly significant from d2 (*P*<0.01). T6FA (2 mg/kg) showed the same effect at the last two days. In the probe trial, swimming times within the target quadrant of mice in the model group were obviously less than those in the sham-operated group (Figure 6 B). However, the shortened swimming time within the platform quadrant induced by Aβ was increased by the treatment of T6FA (2 mg/kg and 20 mg/kg). No significant difference was found between donepezil treatment and high dose T6FA treatment (Figure 6 B).

### T6FA increases ChAT activity and decreases AChE activity in Aβ icv mice

ChAT activity and AChE activity were measured after the learning and memory tests. As shown in Table 2, compared with the sham-operated group, the ChAT activity was significantly decreased in model group (*P*<0.05), whereas the T6FA and donepezil treatments dramatically reversed this change. In contrast, the AChE activity was significantly elevated in hippocampus in the model mice, which was reversed to normal levels of mice in sham group.

**Table 2:** Effects of T6FA on ChAT and AChE activity of in hippocampus of Aβ_1–40_ icv C57 BL/6J mice.

Groups	ChAT (U/g protein)	AChE (U/mg protein)
Sham	94.84±11.22	0.13±0.005
Model	30.00±5.95*	0.18±0.007**
T6FA (2 mg/kg)	73.63±6.55##	0.12±0.010##
T6FA (20 mg/kg)	61.41±7.68#	0.13±0.012##
Donepezil (5 mg/kg)	59.39±8.74#	0.13±0.005##

Data were presented as mean ± S.E.M. n = 10;

*P<0.05,

**P<0.01 compared with sham-operated group;

# *P*<0.05,

# # *P*<0.01 compared with model group.

### T6FA decreases the oxidative stress in Aβ icv mice

To evaluate the effects of T6FA on the oxidative stress in Aβ icv mice, SOD activity and MDA level, two factors indicating the oxidative stress, were also assayed after the MWM test. The SOD activity in model group was significantly lower than that in sham-operated group (*P*<0.05), while the SOD activity was significantly increased (*P*<0.05) by T6FA (2 mg/kg) (from 127 to 161 U/mg protein). Interestingly, the effects of T6FA (20 mg/kg) was milder (*P*<0.01, Table 3). In contrast, the MDA level was significantly higher in hippocampus in model mice than that in sham-operated group (*P*<0.05). All the treatments markedly reversed the increased MDA level (*P*<0.01, Table 3).

**Table 3:** Effects of T6FA on SOD activity and MDA level in hippocampus of C57 BL/6J mice by the i.c.v. injection of Aβ_1–40_.

Groups	SOD (U/mg protein)	MDA (nmol/mg protein)
Sham	154.93±8.62	0.66±0.09
Model	127.10±7.49*	1.05±0.10*
T6FA (2 mg/kg)	161.10±7.21#	0.72±0.14#
T6FA (20 mg/kg)	139.18±7.43	0.70±0.08#
Donepezil (5 mg/kg)	168.03±8.26##	0.58±0.07##

Data were presented as mean ± S.E.M. n = 10;

*P<0.05,

# *P*<0.05,

# # *P*<0.01 compared with model group.

## Discussion

In the present study, we evaluated the multiple-potent effects of T6FA against AD both *in vitro* and *in vivo*. Our in vitro results demonstrated that T6FA significantly inhibited Aβ aggregation induced by AChE, and blocked the cell death and the intracellular ROS accumulation induced by Aβ in PC12 cells. Moreover, we also observed that T6FA significantly improved the cognitive impairment, increased ChAT and SOD activity, and decreased AChE activity and MDA level in Aβ i.c.v. AD model.

Diverse lines of evidence suggest that Aβ plays a causal role in the pathogenesis of AD, the most frequent neurodegenerative disorder and the most common cause of dementia in the elderly [32]. Prefibrillar oligomers of the Aβ are recognized as potential mediators of AD pathophysiology. Increasing evidence showed that AChE is one of the several proteins associated with Aβ aggregation and amyloid plaque deposits [31], [33], [34]. Recently, novel dual inhibitors of AChE that target both the catalytic site as well as the peripheral anionic site (PAS) were found to prevent the aggregation of Aβ into Alzheimer's fibrils [35], [36]. Our previous research found that novel compound T6FA could inhibit AChE through interacting with the catalytic site and PAS simultaneously [17], [19], suggesting that T6FA might inhibit AChE-induced Aβ aggregation. Here we demonstrated that T6FA dramatically inhibit AChE-induced Aβ aggregation by 50.27% and 20.23% at 100 µM and 50 µM, respectively (Table 1). In addition, phenolic compounds, including FA, can inhibit the Aβ auto-aggregation [13]. So, we further investigated the effects of T6FA on the auto-aggregation of Aβ. We found that T6FA (10–25 µM) remarkably inhibited the auto-aggregation of Aβ (Figure 3). Given the toxicity of Aβ is mainly mediated by the prefibrillar oligomers of Aβ and Aβ-induced ROS [5]–[8], we hypothesized that T6FA could block the Aβ-induced cell death through its anti-oxidant activity and anti-aggregation.

In the present study, we have shown that in PC12 cells, T6FA (2–50 µM) prevents the neurotoxicity induced by Aβ_1–40_, a proteolytic derivative of the large transmembrane amyloid precursor protein, which plays a crucial role in AD (Figure 4A). Free-radical oxidative stress, particularly of neuronal lipids, proteins and DNA, is extensive in those AD brain areas in which Aβ is abundant and even those mild cognitive impairment brains [5]–[8], [37]. Our previous research demonstrated that T6FA has antioxidant activity determined by scavenging stable DPPH radicals [17], [19]. DPPH radicals are widely used for the preliminary screening of compounds capable of scavenging activated oxygen species since they are much more stable and easier to handle than oxygen free radicals [38], [39]. Aβ was reported to significantly increase intracellular ROS [5]–[8], [37]. In the present study, Aβ_1–40_ significantly induced ROS accumulation in PC12 cells and the effects can be reversed completely by the pre-treatment of T6FA at 10 µM (Figure 4B).

Clearly, T6FA has protective effects against cholinergic deficiency, oxidative stress and Aβ toxicity *in vitro*. However, the protective effects of T6FA *in vivo* were still unknown. Our previous research found that T6FA can improve the scopolamine-induced cognitive dysfunction in mice [17]. These results suggested that T6FA can penetrate the blood-brain barrier into brain. So we further investigated the effects of T6FA in AD mice induced by Aβ_1–40_, which had been evaluated as a validated animal model for anti-AD drugs development [40]. Following the central administration of the synthetic peptides Aβ_1–40_ analogous to peptides found in neuritic plaques of AD patients, cognitive deficits emerge in mice [26]. In our study, T6FA significantly reduced the escape latency during the hidden platform sessions and also increased the time in the target quadrant as compared with model group (P<0.05) (Figure 6), indicating that T6FA could ameliorate the impairment of learning and memory following Aβ_1–40_ administration.

We also measured the inhibitory effects of T6FA on cholinergic systems and anti-oxidative activity in AD mice. Our results showed that T6FA could significantly increased ChAT and SOD activity, decreased AChE activity and MDA level, indicating it also has ability of antioxidation and inhibiting cholinergic deficiency *in vivo* (Table 2 and Table 3). Meanwhile, we found that no significance was observed in the high and low doses of T6FA (20 mg/kg and 2 mg/kg) while the some better effects on the activities of ChAT and SOD were observed in 2 mg/kg group, which maybe due to the fact that T6FA might down-regulate the SOD expression at the high dose. But further studies should be carried out to uncover it, such as using RT-PCR or western blotting to detect the levels of mRNA/protein of SOD. Further studies should be carried out to answer the question.

In addition, increasing evidences indicated that AChE inhibitors, such as tacrine [41] and rivastigmine [42], lower the amyloid protein *in vitro* independent of their AChE inhibitory. It should be very interesting whether T6FA can also modulate the level of amyloid protein or not. Future studies are needed to be done to discover the question *in vitro* and *in vivo*.

Taken together, the present study demonstrated that, T6FA, a new tacrine-ferulic acid heterodimer, potently inhibit auto- and AChE-induced aggregation. Further, the findings that T6FA blocks or prevents Aβ_1–40_ induced cell death and ROS *in vitro* and chronically oral administration of T6FA protected mice against Aβ_1–40_-induced cognitive impairment *in vivo* strongly suggest that T6FA is a novel “one-compound-multi-targets” agent and might be useful as preventive and therapeutic medicines for AD. Additional *in vitro* and *in vivo* studies in other models to uncover the anti-AD effects of the novel compound are currently underway in our laboratory.
